# How Plan Analysis can inform the construction of a therapeutic relationship

**DOI:** 10.1002/jclp.23300

**Published:** 2022-01-17

**Authors:** Ueli Kramer, Shimrit Fisher, Sigal Zilcha‐Mano

**Affiliations:** ^1^ Department of Psychiatry, Institute of Psychotherapy and General Psychiatry University Hospital Center and University of Lausanne Lausanne Switzerland; ^2^ Department of Psychology University of Windsor Windsor Canada; ^3^ Department of Psychology University of Haifa Haifa Israel

**Keywords:** case formulation, case study, motive‐oriented therapeutic relationship, personalizing, Plan Analysis, psychodynamic psychotherapy, therapeutic relationship

## Abstract

The construction of a positive therapeutic relationship was shown to be related with outcome in psychotherapy, but there are only a few prescriptive concepts helping the therapist to contribute to such a process. The present case illustrates the use of Plan Analysis (PA) and the motive‐oriented therapeutic relationship (MOTR) in the explanation of the construction of a positive therapeutic relationship. We analyze the case of Sharon, a 22‐year‐old student presenting with major depressive disorder. We present the case formulation according to PA and select Session 7 from the therapeutic process to illustrate three moments of the therapist focus on the underlying motives: (a) a first moment when the therapist presents with nonoptimal features of responding to the patient's profile, (b) a second moment when the therapist intervenes optimally, and (c) a third moment when the therapist intervenes excellently. We discuss this case from the perspective of personalizing psychotherapy.

## 1 INTRODUCTION

The quality of the therapeutic relationship substantially contributes to the outcome of psychotherapy, yet it is still difficult to describe in detail what a therapist should do to develop a good quality of the therapeutic relationship. Also, the number of factors influencing the quality of the therapeutic relationship is large, so it is a difficult task to take all these factors into account on the level of the clinical intervention. Among these factors are interpersonal patterns (Benjamin, 1993; Luborsky & Crits‐Christoph, 1998). A recent study has shown that for patients with personality disorders, the presence of certain types of problematic interpersonal patterns, as conceptualized by the core conflictual relationship theme (CCRT), moderated the therapy outcomes in significant ways (Hegarty et al., 2020).

To offer the therapist a guide to the complexity of factors potentially influencing the quality of the therapeutic relationship in the individual case, a case formulation may help (Eells, 2007; Kramer, 2019). Generally, case formulation aims at synthesizing relevant information about the origins of the patient's problems and relationships. The process of developing a case formulation starts at the beginning of psychotherapy and will generally continue, as a therapist‐centered mindful activity, all throughout the treatment, as the formulation itself gets updated with new emerging information from the process of therapy (Eells, 2007): it remains an open question whether that updating describes a new observation by the therapist of something that has been there since the beginning or whether that updating describes an emergent patient manifestation in the process. Case formulation helps to integrate the patient's clinical features within a coherent explanation informed by the clinical theory. Case formulation may be the basis for treatment planning (i.e., which interventions should a therapist use for a particular patient) and case formulation may inform moment‐by‐moment responsiveness to patient manifestations and support tailored relationship building (Caspar, 2021).

Research on case formulation is still in its infancy (Kramer, 2020). Since Persons' (1991) call for a shift in paradigm toward the study of individually‐informed theory‐based mechanisms of psychotherapy, only a handful of studies have addressed the question of the effects of case formulation in psychotherapy. For a sample with patients with bulimia, Ghaderi (2006) showed small to moderate outcome advantages for patients who received a treatment that was informed by a case formulation, as compared to patients who received a treatment uninformed by a case formulation.

Therapist expertise was explored as reflected in the quality of case formulation in a number of studies (Silberschatz, 2013). Eells et al. (2005) compared experts with experienced and novice therapists in terms of precision, content coherence, comprehensiveness, elaboration, and complexity of their case formulations. The results showed that experts made more comprehensive, elaborated, and complex formulations than the other two groups of therapists. It was shown that case formulation contributed to an individualized—idiographic—construction of a positive therapeutic relationship (Gazillo et al., 2019; Kealy et al., 2020; Silberschatz, 2013), in particular, it was shown that case formulation may foster therapist responsiveness from an individualized perspective (Caspar, 2019; Kramer, 2021).

While different methods of case formulation are effective in synthesizing information and providing guidance for the development of a treatment Plan, case formulation also assists the practicing clinician in construing a positive therapeutic relationship, in particular, in cases when interpersonal patterns are prominent features, and may potentially interfere with effective treatment. Plan analysis (PA) is a case formulation method that may be used to this effect. A predecessor of PA, vertical behavior analysis, has been developed by Grawe (1980), originally from clinical experience with behavioral group therapy for social phobia and other clinical presentations, utilizing concepts from general psychology (Miller et al., 1960; following their tradition, Plan is written with a capital P to highlight that the meaning of Plan differs from everyday language by including nonrational, nonconscious strategies); there is also a difference with its use in control‐mastery theory (Silberschatz, 2013), although also notable parallels.

In the clinical context, Grawe (1980) observed that certain patients did not optimally benefit from treatment, despite its technically correct and adherent delivery to behavior therapy principles, so more idiosyncratic aspects linked to these cases were considered useful. PA conceptualizes such idiographic aspects by referring to instrumental links between the patient's observed behaviors and experiences and (inferred, behavior‐underlying) Plans. The latter are understood as means to attain goals and are also understood as the motives or goals themselves explained by the means serving them (Caspar, 2007, 2019). As such, a specific Plan may serve several higher‐level motives which may serve again as Plans (or means) for even higher‐level motives. A specific plan may represent the motive for several means (i.e., lower‐level plans) or observed patient behaviors. A hierarchical structure linking behaviors to underlying motives results. For example, an in‐session behavior, by a patient, of criticizing the therapist with harsh words may serve the plan “present as a nasty person” or “make sure to have the therapist attention,” or “try to show your aggressiveness.” For such a patient, the purpose of presenting as nasty person may be to receive special treatment, or to “make sure the therapist is particularly engaged in this treatment,” which may again serve an even upper‐level Plan of “make sure to have the best treatment,” which may again be related with both “maintain an image of yourself as strong” and “commit to treatment” (which may be related with higher‐order needs to receive healing, for example).

Once the case is formulated in the form of a synthesized Plan structure, the therapist may then select specific Plans to proactively support and intervene by focusing on the underlying acceptable motive. It is assumed that going up in the hierarchy of Plans, one arrives sooner or later—even for a very problematic behavior—at a guiding motive that is acceptable in the sense of not unduly limiting a therapist in their actions or overstraining the therapist. It is assumed that such a focus fosters the quality of the therapeutic relationship (Caspar, 2019; Grawe, 1992). In the example above, the therapist may proactively act complementarily—or in a motive‐oriented way—to a Plan such as “make sure to have the best treatment,” while it may not be advisable for the therapist to be complementary to “present as nasty person.” The latter Plan is understood here as a means that serve the (acceptable) motive of “make sure to have the best treatment”: the means are problematic, but the underlying motive is acceptable. For example, the therapist may say here (to be oriented towards the underlying motive, but not the problematic means): “It sounds like it is so important to you to have the best possible treatment. This is a legitimate demand, and I will do everything possible to make that happen.”

The motive‐oriented therapeutic relationship (MOTR) involves the therapist to select a level on the Plan structure that is sufficiently close to the concrete behavior (ensuring a truly idiosyncratic relationship building), while at the same time avoiding to reinforce the lower‐level Plans (i.e., the problematic means), which may, once activated, interfere with the quality of the therapeutic relationship. The motto is “as high in the hierarchy (of the Plan structure) as necessary, but as low as possible”: this motto yields a therapist intervention that is generally focused on a patient Plan in the middle of the PA (i.e., neither focused on a general need like “stay in control,” nor on a problematic means like “express hostility to the therapist,” but on a Plan like “show that your problems need particular attention,” for example). It is assumed that such a direct focus on the acceptable motives may free the therapist of resources otherwise bound by relationship concerns in favor of work on the patient's central problems, may increase the chance the patient deems this therapist as credible and trustworthy and may offer the conditions necessary for the patient to make corrective relationship experiences.

The procedures detailed above are described in a number of publications (e.g., Caspar, 2007, 2019, 2021) which explain the methodology of using PA and the MOTR in clinical practice and for research. Training is necessary that involves practice‐based exercises on clinical cases, and role‐plays. For research, inter‐rater reliabilities of the different steps of the elaboration of a patient's Plan structure and of the assessment of the MOTR are established; results are generally excellent after sufficient training (Caspar et al., 2005).

Research has shown that such a focus on the (acceptable) motives guiding even problematic behavior may be productive for patients with borderline personality disorder. In a randomized controlled trial, one group of patients received standard brief treatment, the other an enhanced treatment where the therapist explicitly used the MOTR, based on an individualized PA (established after Session 1 into the treatment). The results showed, similarly to Ghaderi (2006) reported above, small to moderate increases in outcome in the end of treatment (Kramer et al., 2014); in addition, the patients who received a treatment where the therapist made a PA and used MOTR reported a stronger increase in the therapeutic alliance over the course of treatment, compared to those who received a treatment without these components (Kramer et al., 2014). It appears from this randomized controlled trial that while PA and MOTR can easily be differentiated on a technical level (i.e., formulate a case without using this information for intervention), from a clinical viewpoint, both concepts must be used together, as MOTR represents the clinical implication of the Plan analytic case formulation. While the therapy in this case study was informed by the MOTR principles based on the Plan‐analytic case formulation, it remains unclear whether in other types of treatment, a post‐hoc formulation of the observed processes may inform the construction of the therapeutic relationship.

The aim of the present case study is to illustrate on a moment‐by‐moment verbatim level the notion of MOTR, based on the case formulation done with the PA in a therapy that was not informed by this conceptualization, similar to the study by Caspar et al. (2005). As such, we want to illustrate patient‐Plan‐compatible therapist behaviors that are linked with the development of a positive therapeutic relationship.

## 2 CASE ILLUSTRATION

The case study illustrated here is taken from a larger ongoing psychotherapy trial in Israel offering free short‐term psychodynamic treatment at the psychotherapy research lab clinic for patients 18–60 years of age, diagnosed with major depressive disorder (of note: specific information were altered in this presentation to protect the patient's identity, as per protocol). In this study, patients received 16‐ to 50‐min psychodynamic psychotherapy sessions using comprehensive treatment protocols. The time‐limited psychodynamic therapy is adapted for depression, either in a supportive expressive‐focused condition (including expressive techniques, such as interpretation, confrontation, clarification; Luborsky, 1984) or a supportive‐focused condition (including the use of supportive techniques, such as affirmation and empathic validation). The supportive condition includes all supportive techniques detailed in the manual used by Luborsky (1984) but forbids the use of any expressive techniques. A detailed trial protocol appears elsewhere (Zilcha‐Mano et al., 2019). The patient was selected out of those diagnosed with personality comorbidity and showed a clinically relevant decrease in symptoms.

Sharon is a 22‐year‐old single woman, a nursing sciences student. She is the youngest of three children to divorced parents. Sharon describes herself as an excellent pupil and self‐disciplined as a child “I was the dream of every teacher,” but she was moody as a teenage girl. Based on her descriptions during the sessions, she devotes most of her time to her studies and work and has only a few close friends.

### 2.1 Presenting problem

Since her early adolescence, Sharon has suffered from recurrent depressive episodes. The first episode occurred at the age of 15, after breaking up with her boyfriend. The breakup was surprising to her, and after a week, she found out that the boyfriend was already in a new relationship. She felt lonely, rejected, and unloved. Her parents, who were busy with their marital crisis, were not available to help her. She was angry with them for blaming her for not recovering and believed that no one could alleviate her pain. Feeling helpless made her bury her feelings and later also adopt a cheerful façade. Although she had therapy sessions, Sharon felt that they were not successful for her. Like with her parents, she was hesitant about sharing her mind openly with her therapist and instead adopted a graceful façade. But inwardly, she constantly felt lonely and alienated. In the past year, her depression has been so intense relative to previous episodes that she thought she had no choice but to seek treatment.

### 2.2 Type of treatment

The treatment framework at the research clinic was supportive‐expressive psychodynamic psychotherapy based on the CCRT (H. E. Book, 1998; Luborsky, 1984) formulation, which focuses on conceptualizing the patient's primary psychodynamic conflicts. During the first few sessions, the therapist assembled the CCRT and then presented it to the patient at the fourth session as a basis for discussion. It is expressed concisely to contain the patient's *wish* in the context of a relationship (W); the patient's anticipated *response from other* in the context of this wish (RO), and the patient's subsequent behavioral and affective *response from self* (RS). The therapist is offering the patient a distilled, well‐integrated narrative (based on the patient's own words) that can direct the therapeutic process. Because it is articulated in the patient's own words and perspective, it helps uncover the patient's unconscious motivations underlying maladaptive behaviors (H. Book, 2004). Based on the CCRT, the therapist built the following case formulation for Sharon: Sharon desired others to understand her genuinely and validate her subjectivity (W). But she was afraid that if others got to know her, they would think she was oppressive and reject her (RO). Therefore, she avoids close relationships. She keeps being blithe (behavioral RS) but feels lonesome (emotional RS). With this formulation in mind, the therapeutic process focused on exploring Sharon's fears, past experiences, and expectations within relationships in the safe environment of psychotherapy. For example, Sharon described several situations in which she hid her authentic feelings from a close friend and explained: “If I'll tell her how I genuinely feel and think she would not like to stay a friend of mine. I wouldn't want a friend like me.” Such statements enabled working through Sharon's wish for confirmation while acknowledging her avoidance of close relationships.

### 2.3 Characteristics of the therapist

Betty is a licensed clinical psychologist in her early forties. She has over 10 years of clinical experience working with depressed patients. Before her enlistment to participate as a psychologist in the study, she attended a 20‐h training workshop in the relevant expressive techniques, such as interpretation, confrontation, and clarification, as well as supportive techniques, such as affirmation and empathic validation.

### 2.4 The first sessions

Sharon presented a significant contrast between her external appearance and behavior and her emotional state. At the first therapy session, Sharon entered the therapy room wearing a light blue dress and a smiling face, which she maintained throughout the session. She talked fast and excessively, using somewhat lighthearted mannerisms while talking, telling Betty how sad and distressed she felt. In the fourth session, when Betty delivered the CCRT and noted this gap, Sharon felt reassured and relieved. She reported feeling understood for the first time: “Oh! Finally! someone is listening to me and understands what I am trying to say!”

### 2.5 Method of case formulation for the present illustration

The PA of this patient was established based on the video material from the first session and followed Caspar's standard recommendations (2019). The MOTR Scale (Caspar et al., 2005) was applied to the seventh session. The MOTR‐rating was done in three steps: (1) definition of the therapist intervention sequence to be rated (i.e., details of the sequencing method described by Caspar et al., 2005), (2) identification of the acceptable Plans (maximum three per sequence, as formulated by the PA) addressed by the therapist in this selected sequence, (3) rating of MOTR (based on the acceptable Plans identified under (2) on two aspects, (a) verbal and (b) non/para‐verbal, on a seven‐point Likert‐type scale (theoretical range between −3 “not motive‐oriented at all” over 0 “neutral” to +3 “absolutely motive‐oriented”). Positive numbers indicate high therapist orientation toward the underlying motive with regard to the patient's currently activated Plan; negative numbers indicate low therapist orientation towards the underlying motive.

Session 1 was selected to develop the PA in keeping with regular practice using PA. Session 7 was selected based on an eye‐ball examination of self‐rated depression scores of all the sessions. Sharon entered treatment with a high depression score which dropped significantly below the clinical threshold in Session 7 and would continue to drop with the Session 8 rating. This drop in depressive symptom around Session 7 suggested that there are processes involved in that session that are particularly productive in reducing depression, which was one of major clinical targets in this treatment. Although there is fluctuation in her level of depression later on, at the end of the treatment Sharon shows very few symptoms. This trend continues into the follow‐up one year later. An expert in the rating procedures (and first author) formulated the PA and rated the MOTR Scale. Regular meetings were held among the co‐authors to discuss the results of the case study.

## 3 CASE FORMULATION USING PA

**Figure 1:**
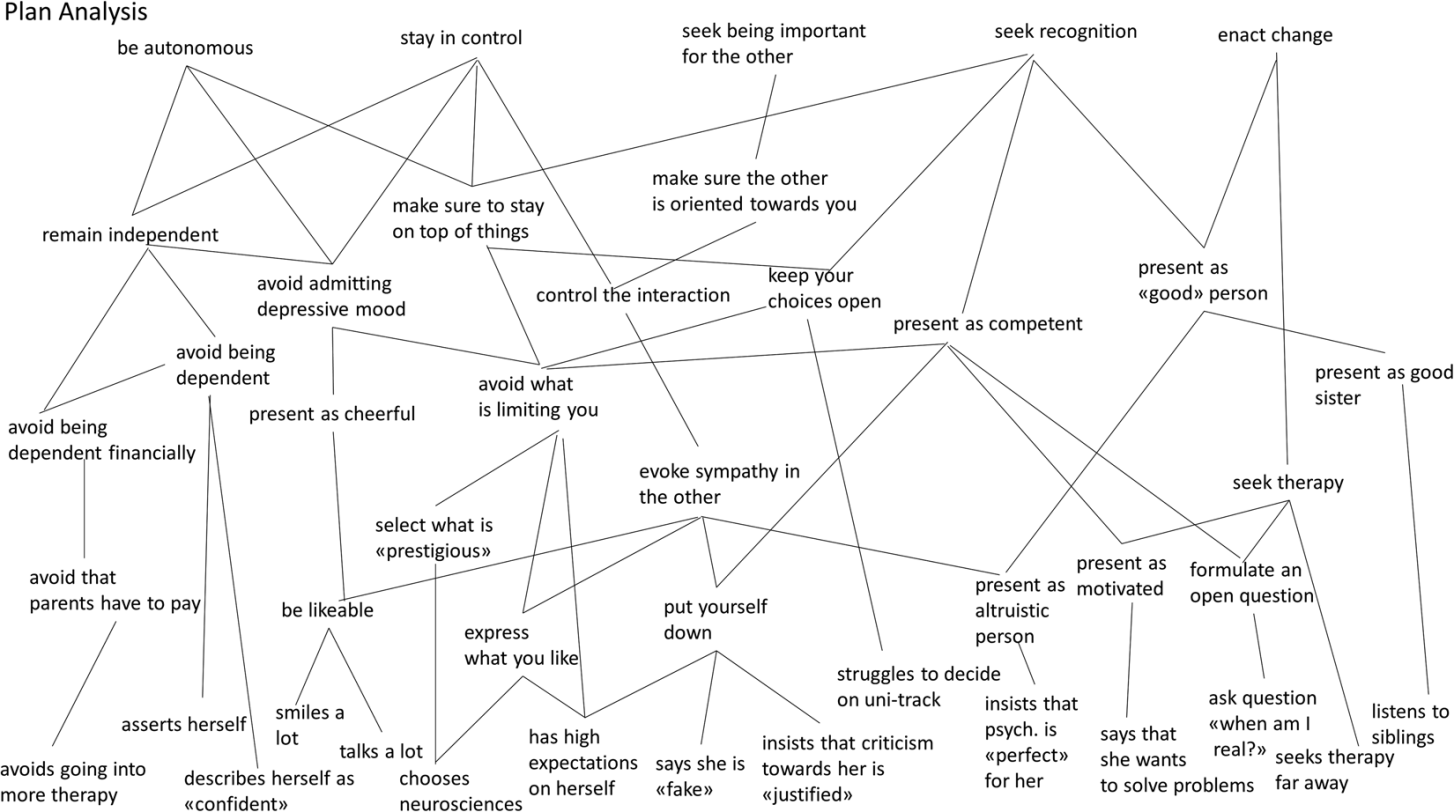
Sharon's Plan Analysis

In what follows, we will comment on the Plan structure by Sharon (see Figure 1), and how it may inform therapeutic decision‐making, then illustrate the therapist use of the MOTR, by drawing on material from Session 7, as based on the PA. For the case of Sharon, we selected 15 behaviors (see Figure 1, at the bottom, are displayed the observed behaviors, in the largest sense) from the first session which we deemed as instrumental. We then determine possible Plans that explain these behaviors, by answering the question of what the purpose of a specific patient behavior may be. These behaviors involve verbal and nonverbal in‐session (and potentially also out‐of‐session) manifestation (as well as information gathered about the patient from assessments). We use here a descriptive language that summarizes several excerpts from Session 1: for example, “talks a lot” may be observed at a number of instances of this session, as well as the observation that Sharon “displays high expectations of herself” (e.g., at one moment during Session 1, the patient said “I want to excel at everything”). We also observed that this patient “smiled a lot,” even during the activity of re‐scheduling a session, then commented that she was sad while smiling. Finally, another example of a behavior that entered into the Plan structure was the instance when Sharon mentions she is “fake,” which she only mentioned once, but it was deemed a sufficiently strong expression with an instrumental component that entered the Plan structure.

Once we had selected the behaviors to be explained, we started inferring the Plans by answering the question of what the purposes, or goals, may be behind each behavior, by staying pragmatically as close to the clinical material possible. Here, we used 28 formulated (i.e., inferred) plans (with five very general motives on the top of the Plan structure: “be autonomous,” “stay in control,” “seek being important for the other,” “seek recognition,” and “get healed”).

It appears that the PA of this patient is marked by a number of avoidance Plans, as can be seen on the left side of Figure 1. Sharon has several means to “stay independent,” such as avoid being dependent financially (which is substantiated by her avoiding to go into more therapy which is paid for by her parents), and such as avoid being dependent (more generally) which is substantiated by behaviors like self‐assertion and her describing herself as “confident” as well as avoid admitting that she has depressive bouts. For the latter, the patient has several means at her disposal that regulate her behavior and experience, for example, she avoids anything that may be limiting herself, or her career, by selecting “prestigious” study topics (e.g., medical sciences), by expressing what she seems to really like and by formulating high expectations toward herself (which may serve ultimately the upper Plan of “avoid admitting depressive mood,” but also the upper Plan “make sure the other is oriented towards you”).

Another important “branch” of the PA is structured around the Plan of “control the interaction.” Again, we can have a look at Figure 1 to see which behaviors and experiences, and lower‐level Plans this person has as mean to fulfill this Plan. One can see that Sharon may evoke sympathy in others by putting herself down, or more specifically by saying “I am fake,” by insisting that criticism towards her is “justified” and by formulating high expectations towards herself.

Finally, there are a number of instrumental links in Sharon's PA that are organized around the Plan “present (yourself) as 'good' person”: its means are her presentation as altruistic (in particular that nursing is the “perfect” discipline of study for her) and her presentation as helpful older siblings.

A MOTR for this patient may take into account this complexity and aim at fulfilling, in the therapeutic process, the behavior‐underlying (generally upper‐level) acceptable Plans (such as “present as good person”, “make sure the other is oriented towards you,” “make sure to stay on top of things,” and/or “remain independent”).

## 4 COURSE OF TREATMENT: THE MOTR IN ACTION

We observe clinically that towards the end of the treatment, Sharon reported an improvement in depressive symptoms. She began expressing her frustrations and needs authentically, realizing that others might respond appropriately to her and sometimes might not. Now she felt she could bear it without feeling rejected and further enjoyed an improvement in close relationships.

**Figure 2:**
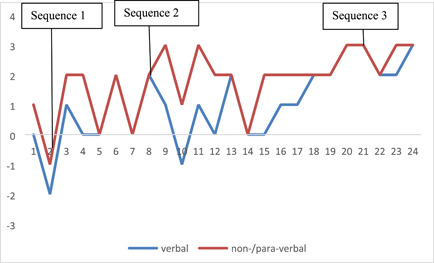
Verbal and nonverbal motive‐oriented therapeutic relationship over the course of Sharon's Session 7

What happened in Session 7 was an example of the psychotherapy process (see the MOTR rating in Figure 2). Plans that were activated in at least two independent rated sequences in this session were: “control interaction” (six instances), “stay in control” (five instances), “make sure to stay on top of things” (four instances), “avoid admitting depressive mood,” and “make sure the other is oriented towards you” (both three instances). It appears that the three first ones are related with the overall need for control, but each describe a facet that is more or less interpersonal. Optimal therapist behavior would have to take these elements of the formulation into account and answer for each Plan what the (acceptable) underlying motive(s) is/are, to orient the intervention in this sense (consistent with MOTR; see below for examples).

## 5 FIRST SEQUENCE (2:56–4:30): THERAPIST STRUGGLING TO BE RESPONSIVE TO THE PATIENT'S GUIDING MOTIVE

This sequence was selected to illustrate a particular low score of verbal and nonverbal complementarity in the sense of the MOTR. The rating is understandable when we consider the observation that the patient's activated Plan here may be “avoid admitting depressive mood,” which according to the PA calls for an intervention oriented toward the motives of stay in control and remain independent/be autonomous. Here is an excerpt where the patient elaborates on her reaction to judgmental comments related with the body (of note, both the patient and therapist appear to be normal‐weighted).

Pt (gesticulating): “(…) to see someone who is fat, and to immediately say 'wow, they are so fat…!' and so on. This really burdened me (touches herself as if she wants to hug herself) at some point.”

Th: “So did you tell them that?” (keeps her arms crossed)

Pt (smiles): … “ehm, no, …. Like, at little. I mean, at some point I did say to my mom (…) that I don't understand why we look at others in such a judgmental way (…). It's like, they will not listen to me, because they all think the same (…). Th (keeps her arms crossed): “mhm.”

Pt (in lively voice): “I feel that (…) my mom and I are distant now (…) for example at the end of the trip I felt we were going at some point and not… we did not say a word, we did not speak about anything. (…) Who is this person who sits in front of me? (…) I did not understand what she was doing. Th (nods): “mhmh.”

Pt: (…) So I began the trip in a very specific stance and I finished it totally differently.”

Th: “In a good or a bad manner?”

Pt: “Not good (therapist nods), and it somehow surprises me, because I can handle these kinds of trips with my family usually …” (moves her legs energetically, as she speaks quickly). Th: nods

Pt: “I don't know… since my parents divorced we keep having this big family vacation once a year—every year.

Th: “Here?”

Pt: “Yes, it's usually here, but I have never felt that…

Th: “Do you have an explanation for it, for this change?”

Pt (looks at fingers and touches them repeatedly): “I don't know …ehm”

Autonomy‐focused interventions, as recommended by the Plan‐analytic case formulation, are hardly visible in the process here; on the contrary, specific interventions made by the therapist, such as (on the verbal level) questions by the therapist, confrontations, and (on the nonverbal level) absence of therapist engagement and retreat behaviors on part of the therapist, might have actually interfered with the patient's activated motives. It appears that facing these activated motives and Plans, in this sequence, the therapist struggles to propose a good level of a MOTR. We may hypothesize that a Plan analytic case formulation may have helped this therapist in this specific sequence to make productive use of the responsiveness in the therapeutic relationship.

## 6 SECOND SEQUENCE (10:18–11.00): THERAPIST MOVING TOWARD A DEEPER RELATIONSHIP OFFER

This sequence was selected to illustrate a moderate to high score of verbal and nonverbal MOTR. The patient's activated Plan was “make sure the other is oriented towards you” (the other may include the therapist in this case). Please note the patient's active part in this formulation of the Plan—“make sure”—which goes beyond a representational wish, but is embedded in potential patient action having an instrumental (goal‐oriented) component. The therapist orients her interventions towards this acceptable motive and constructs a new territory of the therapeutic relationship basis with this patient.

Pt: “She (patient's sister) is at such a phase where she thinks of herself alone…

Th: “You began saying that you also would have wanted a hug.” Patient: “Yea.”

Th: “This is the essence (smiles at patient; voice is strong), I mean, this is your wish.”

Pt: “Yes, yes.”

Th (interrupts): “To receive the support and the hug, the understanding.”

Pt: “I think they did understand me and it was very literal, very verbal, we were all on the same page at the end of the day, except for my sister (…) (speaks very quickly). She needed to have them around her needed to be around (gesticulates showing warmth towards someone), and my dad and my mom approached her. I was so angry; I was really mad at her. My anger was out of place, it was out of context. I was so angry and on the edge.”

Th: “Yeah, (…)

Pt: “and she gets…”

Th: “The hugs and…”

Pt: “yes”

It appears that the therapist is moving toward taking explicitly and implicitly the patient's underlying motive into account, and this without having done a Plan analytic case formulation. This may be because, in this sequence, the patient's activated Plans are more proactive and thus, more easy to use productively in the therapist's responsive interventions.

## 7 THIRD SEQUENCE (39:55–42.33): THERAPIST WORKS WITH “THAT GOOD FEELING”

This third sequence from much later in the seventh session was selected because of the excellent scores on both the verbal and nonverbal MOTR. The therapist orients her interventions explicitly and implicitly toward the two activated Plans of “make sure the other is oriented towards you” and “engage in therapy.”

Pt: Yes so… I am like…

Th: “With her (friend), you felt like she understands you? That she could give you something… To support you, strengthen you?”

Pt: “Yes. Yes, very much.” (looks down). Th: “what?”

Pt: (thinks deeply by looking down) “I think that I felt really… that she does not judge me, and yea, basically and that really was expressed by us having a very stable relationship. We almost never see each other but we do talk to each other (…) I know that every time I see her… it is like a warm home.”Th nods and smiles.

Pt: “A place that I can come back to and it will always accept and love and all that.”

Th: “Why are you not in touch more often?”

Pt: “I was just thinking about it.” (smiles openly) “Because I think that it is only a geographical issue; she lives on one side of the country and I live on the other. And she is a med student, so she really is consumed with her studies.” Th: “mhm.”

Pt: “I mean when we WERE together, we really had an amazing relationships, all the time. And now it seems that…but it has been like this for years now, for two or three years we are like this. And I think it has been fine by me, I mean I don't have to have a very close relations with all the people I love…” Th: “No, that's okay.”

Pt: “Yeah I am telling that to myself.”

Th: “yes I am trying to understand what does she give you that is good for you. So, we might be able to understand what do you want from other people, also… (engaging voice, looks at patient by nodding at her) from the significant others, not everyone.” Pt: “Yes.”

Th: “From significant others, so I am trying to understand what does she give you in this relationship that is good for you, and meets your needs. Not in theory, but to you…” Pt: “Yes.”

Th: “So you said she is not judgmental. She can listen without judging you. Which by itself gives that good feeling.” Pt nods by smiling.

Th: “But in your relationship with your father there is something a bit more judgmental, right? He has his standards, so if you will not meet them, he will tell you.” Pt: “yes.”

Th: “fine (nodding) It is just that, you know, it is not easy to distinguish, to understand what is bothering you there, what in that relationship with your father is less enabling to share… different parts of you. What in your bond with this friend that is enabling you to share…” (uses mimic and gestures to engage)

In this sequence, the therapist had excellent complementarity for both verbal and nonverbal behavior that is consistent with the patient's Plans “engage in therapy” and “make sure the other is oriented towards you.” It is interesting to observe the therapist's owning on some of the complementarity, by the verbal and non‐verbal markers in the transcript. Again, this has been achieved without actually doing a Plan analytic case formulation, but the proactive nature of the activated Plans may help explain the therapist's responsiveness.

## 8 OUTCOME AND PROGNOSIS

As the therapist navigated around the problematic aspects of Sharon's Plan structure, it appears that the therapist conveyed trust in the patient and assured both her difficulties and strengths; therefore, the patient felt safe and relieved. Based on this case and specifically from our observations from the key Session 7, it can be proposed that the therapist's tailoring the treatment to the case may have contributed to further positive changes in symptom reduction right after that session. Whereas the therapist was not familiar with the MOTR while working with this patient, it seems she was able to implement (in particular during the third sequence presented) therapeutic issues that are of great importance both according to the MOTR and supportive‐expressive treatment manual. At the same time, it seemed more difficult for this therapist to use MOTR in the first sequence when the patient's activated Plans concerned issues of control and autonomy (see also Caspar et al., 2005).

## 9 CLINICAL PRACTICES AND SUMMARY

The present case study illustrates the utility of the MOTR in the endeavor of explaining how personalizing psychotherapy to the individual patient may work. In the specific case presented here, we found the therapist complementarity in the sense of MOTR was moderate to high. Even if not intending to do so, the therapist was working, to some extent, with the patient's guiding motives. This may demonstrate the transtheoretical nature of the PA where even when implemented on a case originally treated based on a different conceptualization (CCRT), it can contribute unique insight and shed further light on our understanding of the individual patient. While a full comparison is beyond the scope of the present work, we find that the two conceptualization approaches (CCRT and PA) can be synthesized to achieve a deep understanding of the patient's wishes, needs, motives, and behaviors.

### 9.1 Therapist responsiveness: Different for each patient

According to her PA, Sharon is seeking recognition from others on the one hand, while avoiding feeling dependent and helpless on the other. We assume here that the therapist's understanding of the patient's underlying motives and how they are activated in therapy may have been instrumental in tailoring the therapeutic relationship to the specific patient. Our observation, in particular from the third sequence presented, is that the therapist accurately recognized and underlined what the patient needed, while at the same time underscoring her message nonverbally in a consistent fashion, which helped her remain oriented toward the patient, while avoiding being judgmental.

It can be proposed that MOTR can be instrumental in guiding the therapist on how to become mindful of the full array of the patient's specific clinical features, for example, in the particular case presented here, the patient avoiding expressing authentic feelings. MOTR can further guide the therapist in addressing both verbal and nonverbal modalities of behavior in which the patient's personality profile is manifested with a differentiated and noncontingent response. Using MOTR may, therefore, open up clinically productive avenues of enhancing the potential of therapist responsiveness, for each individual according to their Plan structure, and using it to the benefit of the patient on the level of moment‐by‐moment attunement to the patient's activated Plans in the session. In this case, the therapist's responsiveness not only facilitates the ability of the patients and therapists to achieve a sense of agreement on their work in treatment (e.g., the therapist remains oriented towards the patient, highly responsive to the content) but also to strengthen the emotional bond between them by facilitating a corrective experience.

This case demonstrates how within a randomized control trial, tailoring the use of the protocol techniques to the patient's Plans and motives can lay the ground of a corrective relational experience between the patient and the therapist. This demonstration is in line with the growing interest in personalized treatment, seeking to choose the optimal treatment from those available for each individual and further tailor the techniques used during treatment to the patient's features. It may be especially optimal for Sharon, who could not express her feelings authentically without feeling criticized, to re‐experience her difficulties within the therapeutic relationship in a way that encouraged corrective experience. We saw that MOTR may be a building‐block to explain this process.

The present study aimed to illustrate, at a moment‐by‐moment verbatim level the notion of the MOTR, based on a case formulation done with the PA in a therapy that was not informed by this conceptualization. As such, we identified therapist behaviors that are supposedly linked with the development of a positive therapeutic relationship. Case formulation may be most helpful in understanding and constructing a positive therapeutic relationship in cases where long‐standing interpersonal patterns are present. By labeling the patient's interpersonal wish explicitly “to be understood” and acting to identify the barriers in fulfilling that wish, the therapist used techniques based on a supportive‐expressive protocol that served to even further align with Sharon's specific Plans and motives.

In the future and in research contexts, it is important to demonstrate reliability of the case formulation, as well as the use of emotional processes (Caspar, 2021). A further potential line of development may be the use of machine learning algorithms to make case formulations. Such algorithms can automatically provide codings of sessions after being trained to mimic experienced coders' coding. While it remains a challenge to translate the case formulation methodology fully into a machine, we can conclude so far that manually coded MOTR, based on the PA, remains a viable gold standard of explaining the development of a positive therapeutic relationship in psychotherapy, by differentiating between the actual words spoken and the non‐verbal components of therapist communication.

### 1 PEER REVIEW

The peer review history for this article is available at https://publons.com/publon/10.1002/jclp.23300

## Data Availability

The data that support the findings of this study are available from the corresponding author upon reasonable request.
